# Supplementary material for: Advanced Molecular Characterisation in Relapsed and Refractory Paediatric Acute Leukaemia, the Key for Personalised Medicine
Source: J Pers Med. 2022 May 27;12(6):881. doi: 10.3390/jpm12060881 (PMC9224967; doi:10.3390/jpm12060881)
Supplement: Supplementary file 1 [file jpm-12-00881-s001.zip › jpm-1675599-supplementary/Table S1.pdf]

Supplementary Table S1: Summary of the genes included in the panel version 1 (v.1) and version 2 (v.2) of the panel.

**v.1 - NAME OF THE GENES IN ALPHABETICAL ORDER (91)**
ABL1,ABL2,ADARB2,AKT1,ARID2,ASXL1,ATRX,BCL11B,BRAF,CBL,CDKN2A,CDKN2B,CEBPA,CHD2,CREBBP,CRLF2,CSF1R,CTCF,CTLA4,DNM2,DNMT1,DNMT3A,DNMT3B,EBF1,ECT2L,EED,EGFR,EP300,EPOR,ETV6,EZH2,FBXW7,FLT3,FLT4,GATA1,GATA2,GATA3,HNRNPA1,HNRNPR,IDH2,IGF1R,IKZF1,IKZF3,IL1B,IL6,IL7R,JAK1,JAK2,JAK3,KDM6A,KIT,KMT2C,KMT2D,KRAS,MPL,MSH6,MYC,NF1,NIPBL,NOTCH1,NPM1,NRAS,NSD2,NT5C2NTRK3,PAX5,PDGFRB,PEAK1,PHF6,PTEN,PTPN11,RAD21,RB1,RELN,RET,RUNX1,SETBP1,SETD2,SH2B3,SLC25A6,SMARCB1,SMC1A,SMC3,SRSF2,STAG2,SUZ12,TBL1XR1,TET2,TP53,USH2A,WT1.
**v.2 - NAME OF THE GENES IN ALPHABETICAL ORDER (182)**
ABL1,ABL2,ACPP,ADARB2,AKT1,AKT3,ALK,AR,ARID2,ASXL1,ASXL2,ATM,ATRX,AXL,BCL11B,BLK,BRAF,BRCA1,BRCA2,BTK,CALR,CBL,CCND1,CCNE1,CD19,CD33,CD52,CDK4,CDK6,CDKN2A,CDKN2B,CEBPA,CHD2,CREBBP,CRLF2,CSF1R,CTCF,CTLA4,CTNNB1,DDR1,DDR2,DICER1,DNM2,DNMT1,DNMT3A,DNMT3B,EBF1,ECT2L,EED,EGFR,EML4,EP300,EPHA2,EPHA3,EPOR,ERBB2,ERBB4,ESR1,ETV6,EZH2,FBXW7,FGFR1,FGFR2,FGFR3,FGFR4,FGR,FLT1,FLT3,FLT4,FYN,GAK,GATA1,GATA2,GATA3,HCK,HDAC1,HDAC10,HDAC11,HDAC2,HDAC3,HDAC4,HDAC5,HDAC6,HDAC7,HDAC8,HDAC9,HNRNPA1,HNRNPR,HRAS,IDH1,IDH2,IGF1R,IKZF1,IKZF3,IL1B,IL6,IL6R,IL7R,INSR,JAK1,JAK2,JAK3,KDM6A,KDR,KIT,KMT2C,KMT2D,KRAS,LCK,LYN,MAP2K1,MAP2K2,MERTK,MET,MLH1,MPL,MS4A1,MSH2,MSH6,MST1R,MTOR,MYC,MYCN,NF1,NF2,NIPBL,NOTCH1,NOTCH3,NRAS,NSD2,NT5C2,NTRK1,NTRK2,NTRK3,PARP1,PARP2,PAX5,PDCD1,PDGFRA,PDGFRB,PEAK1,PHF6,PIK3CA,PIK3CD,PTEN,PTPN11,RAD21,RAF1,RARA,RB1,RELN,RET,ROS1,RUNX1,SDHD,SETD2,SH2B3,SLC25A6,SMAD4,SMARCA4,SMARCB1,SMC1A,SMC3,SMO,SRC,STAG2,SUZ12,TBL1XR1,TERT,TET2,TNFRSF8,TNFSF11,TNFSF13B,TP53,TSC1,TSC2,TYRO3,USH2A,VEGFA,VEGFB,VHL,WT1.
**NAME OF THE COMMON GENES IN BOTH VERSIONS v.1 & v.2 (90)**
ABL1,ABL2,ADARB2,AKT1,ARID2,ASXL1,ATRX,BCL11B,BRAF,CBL,CDKN2A,CDKN2B,CEBPA,CHD2,CREBBP,CRLF2,CSF1R,CTCF,CTLA4,DNM2,DNMT1,DNMT3A,DNMT3B,EBF1,ECT2L,EED,EGFR,EP300,EPOR,ETV6,EZH2,FBXW7,FLT3,FLT4,GATA1,GATA2,GATA3,HNRNPA1,HNRNPR,IDH2,IGF1R,IKZF1,IKZF3,IL1B,IL6,IL7R,JAK1,JAK2,JAK3,KDM6A,KIT,KMT2C,KMT2D,KRAS,MPL,MSH6,MYC,NF1,NIPBL,NOTCH1,NPM1,NRAS,NSD2,NT5C2,NTRK3,PAX5,PDGFRB,PEAK1,PHF6,PTEN,PTPN11,RAD21,RB1,RELN,RET,RUNX1,SETBP1,SETD2,SH2B3,SLC25A6,SMARCB1,SMC1A,SMC3,STAG2,SUZ12,TBL1XR1,TET2,TP53,USH2A,WT1.
