# Supplementary material for: Advanced Molecular Characterisation in Relapsed and Refractory Paediatric Acute Leukaemia, the Key for Personalised Medicine
Source: J Pers Med. 2022 May 27;12(6):881. doi: 10.3390/jpm12060881 (PMC9224967; doi:10.3390/jpm12060881)
Supplement: Supplementary file 1 [file jpm-12-00881-s001.zip › jpm-1675599-supplementary/Table S2.pdf]

Supplementary Table S2: Summary of clinical data of the patients

ID	SEX	AGE DX	DISEASE	CNS DX	FIRST TREATMENT PROTOCOL	RISK GROUP DX	CR AFTER INDUCTION	HSCT IN 1st CR	R/r	RELAPSE LOCALIZATION	AGE AT R/r	r/R TREATMENT	EXITUS	CAUSE OF EXITUS	FOLLOW UP (MONTHS)
HRL 1	F	13	MPL	1	LAL-SEHOP-PETHEMA-2013	High	NO [1]	YES	r	-	13	CHEMOTHERAPY (MYELOID SCHEMA) + HSCT	YES	TRM	7,4
HRL 2	F	1	B-ALL	1	LAL-SEHOP-PETHEMA-2013	Intermediate	YES	NO	R	BM	9	CHEMOTHERAPY (LAL-SEHOP-PETHEMA-2015 RECAÍDAS) + HSCT + TRAMETINIB	YES	PROGRESSION	49
HRL 3	M	2	B-ALL	1	LAL-SHOP-2005	Standard	YES	NO	R	BM	11	CHEMOTHERAPY (SEHOP-PETHEMA-2015 RECAÍDAS)	NO	-	154,6
HRL 4	M	8	B-ALL	1	LAL-SEHOP-PETHEMA-2013	Intermediate	NO	YES	r [2]	-		CHEMOTHERAPY (LAL-SEHOP-PETHEMA-2013) + RUXOLITINIB + HSCT	NO	-	42,5
HRL 5	M	13	T-ALL	1	LAL-SEHOP-PETHEMA-2013	Intermediate	YES	NO	R	BM	15	CHEMOTHERAPY (LAL-SEHOP-PETHEMA-2015 RECAÍDAS)	N/A	N/A	-
HRL 6	M	2	B-ALL	1	LAL-SEHOP-PETHEMA-2013	Intermediate	YES	NO	R	Combined (BM and teste)	4	CHEMOTHERAPY (LAL-SEHOP-PETHEMA 2015 RCEAÍDAS) + HSCT	NO	-	63,7
HRL 7	F	12	AML	1	NOPHO-2012	Standard	NO	YES	r and R [3]	BM	12	SALVAGE CHEMOTHERAPY + SUNITINIB + HSCT	NO	-	37,7
HRL 8	F	0	AML	1	LANL-SHOP-2007	High	YES	NO	R	BM	1	SALVAGE CHEMOTHERAPY + HSCT	NO	-	123
HRL 9	M	0	T-ALL	1	INFERFANT-06	Low	YES	YES	R	BM	1	SALVAGE CHEMOTHERAPY + NKS INFUSSION	YES	PROGRESSION	6
HRL 10	M	15	B-ALL	1	LAL-SEHOP-PETHEMA-2013	Intermediate	NO	AWB [4]	r	-	15	SALVAGE CHEMOTHERAPY + RUXOLITINIB + HSCT	YES	TRM	12
HRL 11	M	12	AML	1	NOPHO-2012	High	YES	YES	R	BM	14	SALVAGE CHEMOTHERAPY + QUIZARTINIB* + HSCT	YES	TRM	35,4
HRL 12	M	9	B-ALL	3	LLA del Grupo Cooperativo Nacional de Venezuela (adapted from ALL Dana Farber)	High	YES	NO	R	Extramedullary (CNS)	10	INTRATHECAL CHEMOTHERAPY	NO	-	52,6
HRL 13	M	16	B-ALL	3	LAL-SEHOP-PETHEMA-2013	Intermediate	YES	YES	R	BM	19	CART-19	NO	-	55,4
HRL 14	F	0	B-ALL	1	INFERFANT-06	Intermediate	YES	YES	R	Combined (BM and CNS)	0	CART- 19	NO	-	29,5
HRL 15	M	7	B-ALL	1	AHOPCA-2010	High	YES	NO	R	Combined (BM and CNS)	11	CHEMOTHERAPY (LAL-SEHOP-PETHEMA-2015 RECAÍDAS) + BLINATUMOMAB + HSCT	NO	-	80,3
HRL 16	M	7	T-ALL	2	AHOPCA-2015	High	YES	NO	R	BM	10	CHEMOTHERAPY (LAL-SEHOP-PETHEMA-2015 RECAÍDAS) + HSCT + RIBOCICLIB	YES	TRM	69
HRL 17	F	9	B-ALL	1	LAL-SEHOP-PETHEMA-2013	Intermediate	NO	NO	r	-	9	SALVAGE CHEMOTHERAPY + TRAMETINIB + CART-19	YES	PROGRESSION	10,5
HRL 18	M	5	B-ALL	1	LAL-SEHOP-PETHEMA-2013	Standard	YES	NO	R	Extramedullary (teste)	8	CHEMOTHERAPY (LAL-SEHOP-PETHEMA-2015 RECAÍDAS) + RADIOTHERAPY	NO	-	79
HRL 19	M	3	B-ALL	1	LAL-SEHOP-PETHEMA-2013	Intermediate	YES	NO	R	BM	8	CHEMOTHERAPY (LAL-SEHOP-PETHEMA-2015 RECAÍDAS)	NO	-	92
HRL 20	M	2	B-ALL	1	LAL-SHOP-2005	Standard	YES	NO	R	BM	12	CHEMOTHERAPY (LAL-SEHOP-PETHEMA-2015 RECAÍDAS) + HSCT	NO	-	109,5

HRL 21	F	7	B-ALL	1	LAL-SEHOP-PETHEMA-2013	Standard	YES	NO	R	Extramedullary (skin)	9	CHEMOTHERAPY (LAL-SEHOP-PETHEMA-2015 RECAÍDAS) + HSCT	YES	cGvHD	36,5
HRL 22	M	12	B-ALL	1	LAL-SEHOP-PETHEMA-2013	High	YES	NO	R	BM	18	CHEMOTHERAPY (LAL-SEHOP-PETHEMA-2015 RECAÍDAS) + DASATINIB + HSCT	NO	-	86
HRL 23	F	5	B-ALL	1	Children Boston Hospital and Dana Farber	Low	YES	NO	R	BM	15	CHEMOTHERAPY (LAL-SEHOP-PETHEMA-2015 RECAÍDAS) + HSCT	YES	PROGRESSION	48,5
HRL 24	M	3	B-ALL	1	LAL-SEHOP-PETHEMA-2013	Standard	YES	NO	R	BM	3	CHEMOTHERAPY (LAL-SEHOP-PETHEMA-2015 RECAÍDAS) + HSCT + RIBOCICLIB	NO	-	53
HRL 25	F	0	B-ALL	1	INFERFANT-06	Intermediate	YES	YES	R	BM	1	CHEMOTHERAPY (LAL-SEHOP-PETHEMA-2015 RECAÍDAS) + HSCT	NO	-	19,6
HRL 26	M	6	T-ALL	1	LAL-SEHOP-PETHEMA-2013	Intermediate	YES	YES	R	Combined (BM and CNS)	7	SALVAGE CHEMOTHERAPY + HSCT	YES	PROGRESSION	10

ID: Patient Identity; DX: Diagnosis: CNS Dx: Central Nervous System Involvement at Diagnosis; CR: Complete Remission; HSCT: Hematopoietic Stem Cell Transplantation; R: Relapsed; r: Refractory; M: Male; F: Female; MPL: Mixed Phenotype Leukaemia; B-ALL: B-cell-progenitor Acute Lymphoblastic Leukaemia; T-ALL: T-cell-progenitor Acute Lymphoblastic Leukaemia; AML: Acute Myeloid Leukaemia; CNS-1: absence of blast in cerebrospinal fluid; CNS-2: blasts & < 5 white cells/µL in cerebrospinal fluid; CNS-3: blasts & > 5 white cells/µL in cerebrospinal fluid; BM: Bone Marrow; CNS: Central Nervous System; TRM: Transplant-related Mortality; cGvHD: Chronic Graft Versus Host Disease; AWB: aplasia without blasts.

[1] HLR 1 diagnosed with MPL received as first line induction treatment a lymphoid-polychemotherapy scheme, with a refectory and progressive disease. Then, a second line treatment with a myeloid-polychemotherapy scheme was given, achieving CR. After that, HSCT was performed.

[2] HRL 4 was diagnosed with B-ALL. Conventional induction treatment was started, and after evaluation, patient was diagnosed with an induction failure. In this moment, HLR 4 continued first-line treatment plus ruxolitinib, achieving after that CR. HSCT was then performed.

[3] HRL 7 diagnosed with AML, suffered a refractory disease. Salvage chemotherapy plus sunitinib was given. After that, patient achieved CR and HSCT was performed. A year later, and 4 months after sunitinib discontinuation, HRL 7 suffered a relapse.

[4] HRL 10 underwent to HSCT in aplasia without blast status after salvage chemotherapy because of the high risk of the disease.

*Clinical Trial NCT03793478.
