# Supplementary material for: Advanced Molecular Characterisation in Relapsed and Refractory Paediatric Acute Leukaemia, the Key for Personalised Medicine
Source: J Pers Med. 2022 May 27;12(6):881. doi: 10.3390/jpm12060881 (PMC9224967; doi:10.3390/jpm12060881)
Supplement: Supplementary file 1 [file jpm-12-00881-s001.zip › jpm-1675599-supplementary/Table S3.pdf]

Supplementary Table S3: Summary of the advanced molecular characterization performed in the analysed samples

PATIENT ID	DISEASE	STATUS	KARYOTYPE[1]	REARRAN-GEMENTS	CNAs	GENE OVEREX-PRESSION	VARIANTS IDENTIFIED BY NGS	PANEL VERSION	% BLASTS	VAF	DEPTH	AMP CLASS	TIER
HRL 1	MPL	D	complex	*E2A-other*	Del. in *CDKN2A/B*,<br>and *GBE1*.<br>Dup. in *EBF1*.	*WT1*	***FLT3*** NM_004119.2:c.2516A>G(p.Asp839Gly)<br>***FLT3*** NM_004119.2:c.2503G>T(p.Asp835Tyr)	v.2	55	0.09<br>0.18	1098x<br>1101x	LO<br>O	1 D<br>1 T
HRL 2	B-ALL	R	normal	N/A	-	NO	***KRAS*** NM_004985.4:c.34G>T(p.Gly12Cys)<br>***NT5C2*** NM_012229.4:c.1100G>A(p.Arg367Gln)	v.1	12	0.13<br>0.07	516x<br>676X	O<br>LO	2 T<br>2 P
HRL 3	B-ALL	R	complex	NO	Del. in *CDKN2A/B*,<br>*PAX5*, *MLLT3*,<br>*MTAP*.<br>Dup. in *ABL1* and<br>*NUP214*.	NO	***PTPN11*** NM_002834.4:c.205G>A(p.Glu69Lys)	v.2	33	0.10	1501x	O	2 D
HRL 4	B-ALL	D	normal	NO	-	-	NO	v.1	88	-	-	-	-
HRL 4	B-ALL	r	normal	NO	Del. in *CDKN2A/B*,<br>and *IKZF1*.	*CRLF2*	NO	v.1	52	-	-	-	-
HRL 5	T-ALL	R	N/A	N/A	-	-	***FBXW7*** NM_018315.4:c.1273C>T(p.Arg425Cys)<br>***NOTCH1*** NM_017617.4:c.5033T>C(p.Leu1678Pro)<br>***AKT1*** NM_001014432.1:c.49G>A(p.Glu17Lys)	v.1	19	0.06<br>0.06<br>0.07	1274x<br>901x<br>1366x	O<br>LO<br>O	1 P<br>1 P<br>2 T
HRL 6	B-ALL	R	hypodiploid	NO	-	-	NO	v.2	2	-	-	-	-
HRL 7	AML	D	normal	NO	-	*WT1*	***FLT3*** NM_004119.2:c.2503_2506delinsC, p.Asp835_Ile836delinsLeu<br>***WT1*** NM_024426.4:c.1372C>T(p.Arg458*)	v.2	50	0.35<br>0.42	1175x<br>1325x	O<br>LO	1 T<br>1 P
HRL 7	AML	R	normal	NO	-	*WT1*	***FLT3*** NM_004119.2:c.2503_2506delinsC, p.Asp835_Ile836delinsLeu<br>***WT1*** NM_024426.4:c.1372C>T(p.Arg458*)	v.2	70	0.89<br>0.95	823x<br>1018x	O<br>LO	1 T<br>1 P
HRL 8	AML	R	normal	NO	-	-	NO	v.2	35	-	-	-	-
HRL 9	T-ALL	D	normal	NO	-	-	***PTEN*** NM_000314.6:c.737_738insTGAA(p.Leu247fs)<br>***PTEN*** NM_000314.6:c.388C>G(p.Arg130Gly)	v.1	84	0.06<br>0.66	1037x<br>1017x	LO<br>O	2 T<br>2 T
HRL 9	T-ALL	R	normal	NO	Del. in *CDKN2A/B*,<br>*MLLT3*, *MTAP* and<br>*PTEN*.	*CRLF2*	***PTEN*** NM_000314.6:c.388C>G(p.Arg130Gly)	v.2	71	0.59	884x	O	2 T
HRL 10	B-ALL	D	normal	NO	-	-	***PTPN11*** NM_002834.4:c.226G>A(p.Glu76Lys)	v.2	78	0.29	108x	O	2 D
HRL 10	B-ALL	r	normal	NO	Del. in *PAX5*, *IKZF1*,<br>*CDKN2A/B*.	*CRLF2*	***PTPN11*** NM_002834.4:c.226G>A(p.Glu76Lys)<br>***KMT2D*** NM_003482.3:c.15289C>T(p.Arg5097*)	v.2	65	0.28<br>0.05	289x<br>905x	O<br>O	2 D<br>2 D
HRL 11	AML	R	normal	NO	-	*WT1*	***WT1*** NM_024426.4:c.1137_1141dupACGGT(p.Ser381fs)<br>***KMT2D*** NM_003482.3:c.1349_1350insAA(p.Pro451fs)<br>***FLT3*** NM_004119.2:c.1779_1832dup(p.Glu611fs)[2]	v.2	40	0.29<br>0.20<br>-	1180x<br>1728x<br>-	LO<br>LO<br>O	1 P<br>2 D<br>1 T
HRL 12	B-ALL	R	N/A	N/A	-	-	***KRAS*** NM_004985.4:c.38G>A(p.Gly13Asp)<br>***ATRX*** NM_000489.4:c.4868T>G(p.Leu1623*)	v.2	100	0.50<br>0.97	373x<br>281x	O<br>LO	2 T<br>2 D
HRL 13	B-ALL	R	normal	NO	Del. in *IKZF1*, *BTG1*<br>and X<br>chromosome.	NO	NO	v.2	60	-	-	-	-

HRL 14	B-ALL	D	normal	*KMT2A-MLLT1 (MLL1-ENL)*	No CNVs	NO	NO	v.2	90	-	-	-	-
HRL 14	B-ALL	R	normal	*KMT2A-MLLT1 (MLL1-ENL)*	No CNVs	NO	***MPL*** NM_005373.2:c.1642delG(p.Ala548fs)	v.2	59	0.13	399x	LO	2 D
HRL 15	B-ALL	R	hyperdiploid	*ETV6-RUNX1*	Del. in *ETV6.*	NO	NO	v.2	40	-	-	-	-
HRL 16	T-ALL	R	hyperdiploid	NO	Del. in *CDKN2A/B* locus (*MTAP, PHF6*).<br>Dup. in *AHI1.*	NO	***PHF6*** NM_032458.2:c.903C>A(p.Tyr301*)<br>***WT1*** NM_024426.4:c.1128_1129insGGATATCG(p.Thr377fs)	v.2	54	0.68<br>0.28	1266x<br>1539x	LO<br>LO	1 D<br>2 P
HRL 17	B-ALL	D	normal	*E2A-other*	-	-	***SUZ12*** NM_015355.3:c.456-2A>T<br>***NRAS*** NM_002524.4:c.183A>C(p.Gln61His)<br>***NRAS*** NM_002524.4:c.38G>A(p.Gly13Asp)	v.2	58	0.06<br>0.19<br>0.07	301x<br>1365x<br>1589x	LO<br>O<br>O	2 D<br>2 T<br>2 T
HRL 17	B-ALL	r	normal	*E2A-other*	Del. in *PAX5* and *CDKN2A/B.*	NO	***NRAS*** NM_002524.4:c.183A>C(p.Gln61His)	v.2	65	0.28	1884x	O	2 T
HRL 18	B-ALL	D	normal	*BCR-ABL1*	Del. in *JAK2* and *PAX5.*<br>Dup. in *SHOX, CRLF2* and *CSF2RA.*	NO	NO	v.2	65	-	-	-	-
HRL 19	B-ALL	R	hyperdiploid	*ETV6-RUNX1*	Del. in *ETV6.*<br>Dup. in *SHOX* and *CRLF2.*	NO	NO	v.2	60	-	-	-	-
HRL 20	B-ALL	R	N/A	N/A	Del. in *CDKN2A.*<br>Dup. in *PAX5.*	NO	***KRAS*** NM_004985.4:c.191_192insTGTTAACCAGTA(p.Tyr64_Ser65insValAsnGlnTyr)	v.1	88	0.19	317x	LO	2 T
HRL 21	B-ALL	D	hyperdiploid	NO	-	NO	***NRAS*** NM_002524.4:c.181C>A(p.Gln61Lys)	v.1	95	0.40	226x	O	2 T
HRL 22	B-ALL	D	normal	*BCR-ABL1*	-	-	NO	v.1	81	-	-	-	-
HRL 22	B-ALL	R	normal	*BCR-ABL1*	-	NO	NO	v.2	42	-	-	-	-
HRL 23	B-ALL	R	N/A	N/A	Del. in *IKZF1.*	NO	NO	v.1	93	-	-	-	-
HRL 24	B-ALL	R[1]	hyperdiploid	NO	-	-	***PTPN11*** NM_002834.4:c.182A>T(p.Asp61Val)<br>***MSH6*** NM_000179.2:c.402dupT(p.Asp135fs)<br>***CREBBP*** NM_004380.2:c.4506G>C(p.Trp1502Cys)<br>***NT5C2*** NM_012229.4:c.713G>A(p.Arg238Gln)	v.2	81	0.37<br>0.09<br>0.38<br>0.25	1790X<br>2430X<br>4155X<br>976X	O<br>LO<br>LO<br>LO	2 D<br>2 P<br>2 D<br>2 P
HRL 24	B-ALL	R[2]	normal	NO	-	-	***PTPN11*** NM_002834.4:c.182A>T(p.Asp61Val)<br>***CREBBP*** NM_004380.2:c.4506G>C(p.Trp1502Cys)<br>***CDKN2A*** NM_058195.3:c.319dupC(p.His107fs)<br>***NT5C2*** NM_012229.4:c.713G>A(p.Arg238Gln)	v.2	84	0.42<br>0.46<br>0.08<br>0.46	713x<br>636x<br>1106x<br>856x	O<br>LO<br>LO<br>LO	2 D<br>2 D<br>2 T<br>2 P

HRL 25	B-ALL	D	normal	*KMT2A-other*	No CNVs	NO	NO	v.2	90	-	-	-	-
HRL 25	B-ALL	R	normal	NO	No CNVs	NO	NO	v.2	60	-	-	-	-
HRL 26	T-ALL	D	normal	NO	No CNVs	-	***PHF6*** NM_032458.2:c.121_122insTCCCTTCTTCCTCG(p.Ala41fs)<br>***PTEN*** NM_000314.6:c.696dupA(p.Arg233fs)<br>***PTEN*** NM_000314.6:c.740T>C(p.Leu247Ser)	v.2	77	0.41<br>0.35<br>0.33	299x<br>1389x<br>1580x	LO<br>LO<br>O	1 D<br>2 T<br>2 T
HRL 26	T-ALL	R	hyperdiploid	NO	No CNVs	*CRLF2*	***PHF6*** NM_032458.2:c.121_122insTCCCTTCTTCCTCG(p.Ala41fs)<br>***PTEN*** NM_000314.6:c.696dupA(p.Arg233fs)	v.2	87	0.44<br>0.88	637x<br>1855x	LO<br>LO	1 D<br>2 T

ID: Identity; CVAs: Copy Number Alterations; NGS: Next Generation Sequencing; VAF: Variant Allele Frequency; MPL: Mixed Phenotype Acute Leukaemia; B-ALL: B-cell-precursor Acute Lymphoblastic Leukaemia; T-ALL: T-cell-precursor Acute Lymphoblastic Leukaemia; AML: Acute Myeloid Leukaemia; D: diagnosis; R: relapsed; r: refractory; Del.: deletion; Dup.: duplication; LO: Likely Oncogenic; O: Oncogenic; P: prognostic; T: therapeutic; 1: first relapse; 2: second relapse

[1] Karyotype: Hyperdiploid karyotype is considered if ≥47 chromosomes and hypodiploid if ≤ 45 chromosomes. Complex karyotype is defined by the presence of ≥3 cytogenetic alterations.

[2] VAF and depth are available for SNPs and indels, but not for internal tandem duplications (ITD)
